# Letter to the editor regarding “A prospective randomized study of multimodal analgesia combined with single injection transversus abdominis plane block versus epidural analgesia against postoperative pain after laparoscopic colon cancer surgery”

**DOI:** 10.1007/s00384-024-04601-2

**Published:** 2024-02-11

**Authors:** Cheng-Wen Li, Xin-Yue Li, Fu-Shan Xue

**Affiliations:** Department of Anesthesiology, Beijing Friendship Hospital, Capital Medical University, NO. 95 Yong-An Road, Xi-Cheng District, Beijing, 100050 People’s Republic of China

Dear Editor:

By conducting a small-sample prospective randomized trial of 67 patients who underwent laparoscopic colon cancer surgery, Kitagawa et al. [1] showed that the multimodal analgesia including a single injection transversus abdominis plane block (TAPB) provided a comparable postoperative pain control to epidural analgesia and was superior to epidural analgesia with regard to postoperative urinary retention. However, we have several questions on their methodology and results.

First, available literature indicates that pain levels after colorectal surgery are more severe during activity than at rest [2]. In this study, however, the authors did not clearly describe the patient’s status when postoperative pain level was assessed. We argue that this issue would have confused their outcome of pain assessment.

Second, unlike the recent other works comparing postoperative analgesia efficacy of regional block and epidural analgesia for colorectal surgeries [3, 4], no any non-opioid analgesic was administered in the epidural analgesia group and only scheduled paracetamol was given in the multimodal analgesia group. In fact, the current Enhanced Recovery After Surgery practices for elective colorectal surgery recommend the use of multimodal opioid-sparing analgesia protocols, in which besides regional block or epidural analgesia, a combination of non-opioid analgesics including acetaminophen, dexmedetomidine, ketamine, and nonsteroidal anti-inflammatory drugs is often included. Furthermore, non-opioid analgesics are required to be given either preoperatively or intraoperatively and continued as scheduled dosing postoperatively, unless contraindicated [5]. It is worth noting that non-standard application or insufficient doses of non-opioid analgesics in the comparator groups have been one of the main concerns for the inadequate design of randomized controlled trials that evaluate local analgesic techniques [6]. Given that non-opioid analgesics can significantly improve postoperative pain control by synergistic and additive effects, we argue that different results would been obtained if this study included the regular application of non-opioid analgesics in two groups according to the current Enhanced Recovery After Surgery practices for elective colorectal surgery.

Third, the primary outcome of this study was the need for additional analgesics during postoperative 2 days, and flurbiprofenaxetil was used as an additional analgesic when the postoperative pain score was > 4 or at the patients’ request. It must be pointed out that flurbiprofenaxetil only is a nonsteroidal anti-inflammatory drug for management of mild pain with a VAS of 3 or less and is commonly used as the cornerstone of multimodal opioid-sparing analgesia protocols, rather than rescue analgesic for moderate to severe pain with a VAS of > 4 [6]. Thus, we wonder if using a weak non-opioid analgesic as a rescue analgesic would have resulted in an increased risk of additional analgesic requirements in this study.

Finally, a single injection of TAPB was combined in the multimodal analgesia group, but intravenous infusion of fentanyl was continued until 48 h postoperatively. Typically, a single injection fascial plane block is effective for 8–12 h. That is, the multimodal analgesia group might have applied an opioid-based multimodal analgesia protocol during most of the study period, though total fentanyl consumption was provided. In the light of opioid-related adverse effects on the postoperative recovery of patients, the current Enhanced Recovery After Surgery practices recommend that opioids should be administered only as rescue on an “as needed” basis to achieve patient comfort and facilitate the return of postoperative function. Thus, we believe that this design limitation would have underestimated the postoperative benefit of multimodal analgesia protocol including a regional block. For example, a systematic review comparing regional techniques for pain management after laparoscopic elective colonic resection demonstrates that abdominal wall blocks are as effective as epidural analgesia for pain control but provide a lower hospital stay and a better postoperative recovery [7].

## Data Availability

No datasets were generated or analysed during the current study.
